# Modelling of aflatoxin G1 reduction by kefir grain using response surface methodology

**DOI:** 10.1186/s40201-015-0190-2

**Published:** 2015-05-02

**Authors:** Farzaneh Ansari, Faramarz Khodaiyan, Karamatollah Rezaei, Anosheh Rahmani

**Affiliations:** Bioprocess and Biodetection Laboratory (BBL), Department of Food Science, Engineering and Technology, University of Tehran, 31587-77871 Karaj, Iran; Department of Food Science, Engineering and Technology, University of Tehran, 31587-77871 Karaj, Iran; Department of Food Science and Technology, Standard Research Institute, 1745-139 Karaj, Iran

**Keywords:** Aflatoxin G1, Pistachio nut, Predictive modelling, Kefir-grain, Optimization

## Abstract

Aflatoxin G1 (AFG1) is one of the main toxic contaminants in pistachio nuts and causes potential health hazards. Hence, AFG1 reduction is one of the main concerns in food safety. Kefir-grains contain symbiotic association of microorganisms well known for their aflatoxin decontamination effects. In this study, a central composite design (CCD) using response surface methodology (RSM) was applied to develop a model in order to predict AFG1 reduction in pistachio nuts by kefir-grain (already heated at 70 and 110°C). The independent variables were: toxin concentration (X_1_: 5, 10, 15, 20 and 25 ng/g), kefir-grain level (X_2_: 5, 10, 20, 10 and 25%), contact time (X_3_: 0, 2, 4, 6 and 8 h), and incubation temperature (X_4_: 20, 30, 40, 50 and 60°C). There was a significant reduction in AFG1 (*p* < 0.05) when pre-heat-treated kefir-grain used. The variables including X_1_, X_3_ and the interactions between X_2_-X_4_ as well as X_3_-X_4_ have significant effects on AFG1 reduction. The model provided a good prediction of AFG1 reduction under the assay conditions. Optimization was used to enhance the efficiency of kefir-grain on AFG1 reduction. The optimum conditions for the highest AFG1 reduction (96.8%) were predicted by the model as follows: toxin concentration = 20 ng/g, kefir-grain level = 10%, contact time = 6 h, and incubation temperature = 30°C which validated practically in six replications.

## Background

Aflatoxin G1 (AFG1) is a secondary toxic metabolite of the fungi *Aspergillus parasiticus*. Monitoring data indicate that humans and animals may be exposed to health problems via the ingestion of AFG1-contaminated foods and feeds. This toxin has been identified as a mutagenic agent to humans [1]. The International Agency for Research on Cancer reported that there was sufficient evidence in animals for the carcinogenicity of naturally occurring AFG1 [2]. This kind of aflatoxin also caused liver tumours in experimental animals, but generally at a lower incidence than aflatoxin mixtures (AFs) and/or aflatoxin B1 (AFB1) [3]. The rank order of toxicity of AFs is AFB1 > AFG1 > AFB2 > AFG2 [4]. Therefore, AFG1 is the second most important toxic agent among AFs and reducing its bioavailability is of great interest for human safety.

Pistachio nuts are one of the important foodstuffs with the highest risk of AFs contamination [5]. The maximum legal limit of AFG1, not only in Iran but also in the European Union (EU), has not been specified. The allowed level of AFG1 was established altogether with other AFs (AFB1, AFB2 and AFG2) and expressed as total aflatoxin (AFT) [6,7]. Iran is the primary pistachio nut-producing country, and produced almost 472,097 tons of pistachio in 2012 [8].

Dini and co-workers reported that 23.5% of the Iranian pistachio nuts were contaminated with AFT higher than the maximum tolerated level (MRL) during the years 2009–2011 [9]. Preventive physical and chemical methods have been proposed to detoxify AFs [10-14]. These detoxification methods have many limitations, such as the loss of nutritional value of foodstuffs, undesirable health effects of the by-products, high energy consumption and expensive equipment requirements. Nowadays, biological methods by utilizing isolates of bacteria and yeast are used to reduce bioavailability of AFs in different food commodities [15-18].

Kefir-grains are a symbiotic association of microorganisms well known for their probiotic microflora due to aflatoxin decontamination effects. A number of investigations have already reported the biological degradation of AFs by *Lactobacillus casei* [19], *Lactobacillus plantarum* [20] and *Saccharomyces cerevisiae* [21], which were similar to those in kefir-grain [22]. Studies have shown that detoxification occurs by binding the AFs to the microorganism cell-wall structure and involve the formation of a reversible complex between the toxin and microorganism surface, without chemical modification of the toxin [18,23]. Based on the above-mentioned studies, most of the published investigations are in the field of AFB1 decontamination and despite the adverse effect of AFG1 in human safety, no studies have been reported on the possibility of AFG1 decontamination by kefir grains, none in pistachio nuts.

The purposes of the current study were as follows: (i) to investigate the effect of kefir-grain on reduction of AFG1 in pistachio nuts; (ii) to predict a model for AFG1 reduction by kefir-grain, if it has significant effect; and (iii) to optimize the method by using statistical experimental methods.

## Materials and methods

### Preparation of pistachio nuts

Pistachio nuts were purchased from a wholesaler in Tehran, Iran. The consignments were mixed together and stored in zip-locked plastic bags at 4°C during the experiment. Preparations of subsamples were carried out at the Mycotoxin Laboratory, Department of Food Science & Technology, Standard Research Institute, Karaj, Iran, and the Scientific and Research Laboratory of Farogh, located in Tehran, Iran.

For minimising the subsampling errors in AFG1 analysis, water slurry of pistachio nut samples were prepared in a 1:1 ratio. In order to provide a uniform paste, the mixture was ground using a slurry machine. Finally, each sample (containing: 12.5 gr pistachio nuts kernel + 12.5 gr shell + 25 mL water) was taken for further experiments. The samples were analysed by high-performance liquid chromatography (HPLC).

### Maintenance, activation and treatment of kefir-grains

Kefir grains were obtained from the Microorganism Bank, Department of Food Science, Engineering & Technology, Tehran University. The grains were kept in skimmed milk at room temperature at 23 ± 2°C, and the medium was exchanged for fresh skimmed milk daily to maintain grain viability. In order to increase the kefir-grain biomass, the grains were activated [24] and kept at 4°C for short-term storage [25]. Kefir-grains were then ground using a high speed blender (minimum 6000, rpm) and mixed with an equal amount of water. Three levels of heating treatment (N = non-heated at room temperature; H = heated at 70 ± 2°C using a heating device for 5–10 min; and U = heated at 110 ± 2°C using an autoclave for 10 min) were utilized to obtain the maximum activity of grains.

### Sample preparation

For preparing test samples, each (50 gr) pistachio paste portion was thoroughly contaminated with the working AFG1 solutions (5, 10, 15, 20 and 25 ng/g). After that, the contaminated samples were inoculated with different amounts of homogenised treated-kefir grain (2.5, 5.0, 7.5, 10.0 and 12.5 gr; respectively equal to 5, 10, 15, 20 and 25% pistachio past sample). The samples were shaken well and incubated at different incubation temperatures (20, 30, 40, 50 and 60°C) and contact times (0, 2, 4, 6 and 8 h) while other factors were kept constant.

### Chemicals and reagents

Standard solution of AFG1 was obtained from Sigma–Aldrich Chemical Company (USA). Methanol, n-hexane, sodium chloride, acetonitrile, nitric acid, potassium bromide, phosphate-buffered saline were from Merck, Darmstadt, Germany. To produce mobile phase, water (600 mL), methanol (300 mL), acetonitrile (200 mL), 350 μL nitric acid (4 mol/L) and 120 mg potassium bromide were mixed. All solvents used for the experiments were of either HPLC or analytical grade.

### Extraction and cleanup of residual AFG1

The extraction and cleanup of residual AFG1 was performed using AOAC official method 999.07 with some minor modification [26]. Each sample was extracted using 180 mL of methanol and 50 mL *n*-hexane. The extract was diluted by 130 mL water and then filtered through a glass microfiber filter. Aflatest immune-affinity column (IAC, Faroogh Scientific and Research Laboratory) was utilized to clean up the samples. For activation of IAC, 10 mL of phosphate buffer saline passed through it. Then, 75 mL of the filtrate passed through the IAC at a flow rate of 1 drop per second. The column was washed with 15 mL water and dried by applying vacuum for 10 s. Finally, elution of IAC was performed using methanol in two steps. First, 1500 μL methanol was poured on the IAC and allowed to pass through by gravity. After one min the eluate was collected in a vial and finally diluted by 1500 μL water before being analyzed by HPLC.

### Aflatoxin G1 analysis

Reversed-phase HPLC using post column derivatization involving bromination was applied to determine AFG1 [26,27]. The HPLC system (Waters 2695) was equipped with pump, a Waters 2475 multi-fluorescence detector and a Chromolit C_18_ analytical column (Merck Chemical Company, Darmstadt, Germany, 200 cm × 4.6 mm × 4 μm). An electrochemical PCD system was applied using Farlib® EDC cell (Faroogh Scientific and Research Laboratory) for bromination purposes. The flow rate was set at 2.5 mL/min. The detector was operated at wavelengths of 365 and 435 nm as excitation and emission wavelengths, respectively. The limit of detection for AFG1 determination was 0.1 ng/g.

### Experimental design and statistical analysis

In this investigation, central composite design (CCD) using response surface methodology (RSM) was employed to determine the more significant factors among variables which predicted to have an effect on AFG1 reduction [28,29]. Thus, 31 experimental runs (as shown in Table 1) were designed using different variables: toxin concentration (X_1_), kefir grain level (X_2_); contact time (X_3_); incubation temperature (X_4_). The role of other variables (such as acid-treated kefir-grain) which may affect the reduction of AFG1 did not investigate.

**Table 1:** **Coded levels and actual values of the variables in central composite design**

**Run**	**Design matrix**								**Experimental results**
	***X***_***1***_		***X***_***2***_		***X***_***3***_		**X**_***4***_		**AFG1**
	***C***	***A***	***C***	***A***	***C***	***A***	***C***	***A***	**(%)**
1	0	15	0	7.5	0	4	0	40	92.13
2	−1	10	−1	5	−1	2	−1	30	88.10
3	+1	20	+1	10	+1	6	+1	50	94.85
4	-α*	5	0	7.5	0	4	0	40	86.00
5	+1	20	+1	10	+1	6	−1	30	95.05
6	+1	20	+1	10	−1	2	+1	50	91.45
7	0	15	0	7.5	0	4	-α	20	90.33
8	+1	20	+1	10	−1	2	−1	30	93.15
9	−1	10	+1	10	+1	6	−1	30	93.60
10	−1	10	+1	10	−1	2	+1	50	94.00
11	−1	10	+1	10	+1	6	+1	50	90.30
12	0	15	-α	2.5	0	4	0	40	91.60
13	0	15	0	7.5	-α	0	0	40	90.33
14	0	15	0	7.5	0	4	0	40	92.07
15	−1	10	+1	10	−1	2	−1	30	80.80
16	+1	20	−1	5	−1	2	−1	30	90.35
17	−1	10	−1	5	+1	6	−1	30	94.50
18	+1	20	−1	5	+1	6	−1	30	94.05
19	−1	10	−1	5	−1	2	+1	50	86.40
20	+1	20	−1	5	−1	2	+1	50	91.45
21	−1	10	−1	5	+1	6	+1	50	83.40
22	+1	20	−1	5	+1	6	+1	50	92.15
23	0	15	0	7.5	0	4	0	40	92.93
24	0	15	+α	12.5	0	4	0	40	92.07
25	+α	25	0	7.5	0	4	0	40	93.68
26	0	15	0	7.5	+α	8	0	40	94.33
27	0	15	0	7.5	0	4	+α	60	93.73
28	0	15	0	7.5	0	4	0	40	90.93
29	0	15	0	7.5	0	4	0	40	92.1
30	0	15	0	7.5	0	4	0	40	92.1
31	0	15	0	7.5	0	4	0	40	86.45

*α =2.

Note: AFG1 concentration (X_1_, ng/g); kefir grain treated at 70°C (X_2_, g); contact time (X_3_, h); incubation temperature (X_4_,°C); coded level (C); actual value (A).

Data analysis was performed using the R statistical software (version R-3.0.3). Tukey statistical test was used for comparing the means. Significant factors affecting the response were examined using analysis of variance (ANOVA). The results were fitted with a polynomial equation by a multiple regression technique. Theoretical validation of the model for the predicted values of six replicates under optimal conditions was performed by running the two-sample *t*-test.

## Results and discussion

### Preliminary investigation

A preliminary study was performed to determine the effect of kefir-grain on AFG1 reduction from pistachio nuts. Thus, an experiment was performed on contaminated test samples with a constant concentration of AFG1 (5 ng/g) and kefir-grain (5 gr), against the same test portion without kefir-grain as a control group by holding all other factors at fixed levels. Generally, kefir-grain positively showed significant effect (*p* = 3.05 × 10^−5^) on AFG1 reduction (data not shown).

### Effect of heat-treated kefir-grain on AFG1 elimination from media

To evaluate the ability of heat-treated kefir-grain on AFG1 elimination, the results of three levels of heating (N, H and U) were analysed by the Tukey multiple comparison test (Table 2). The results indicated that although there was no significant difference between H- and U-heat treatment of kefir-grain (treated at 70°C and 110°C, respectively), there were significant differences between these two levels of heating and non-heated grains (N) (*p* < 0.05).

**Table 2:** **Multiple comparisons of variables means on AFG1 reduction**

**Comparison**	**Differences**	**Lower**	**Upper**	**Adjusted-p**
H-N	−60.621	−66.998	−54.245	0.000*
H-U	2.796	−3.579	9.173	0.539
N-U	63.418	57.042	69.795	0.000*

Note: Kefir grains treated in three levels (N = non-heated at room temperature, H = heated at 70°C and U = heated at 110°C.

*Significant at the 5% level (*p* < 0.05).

**Figure 1:**
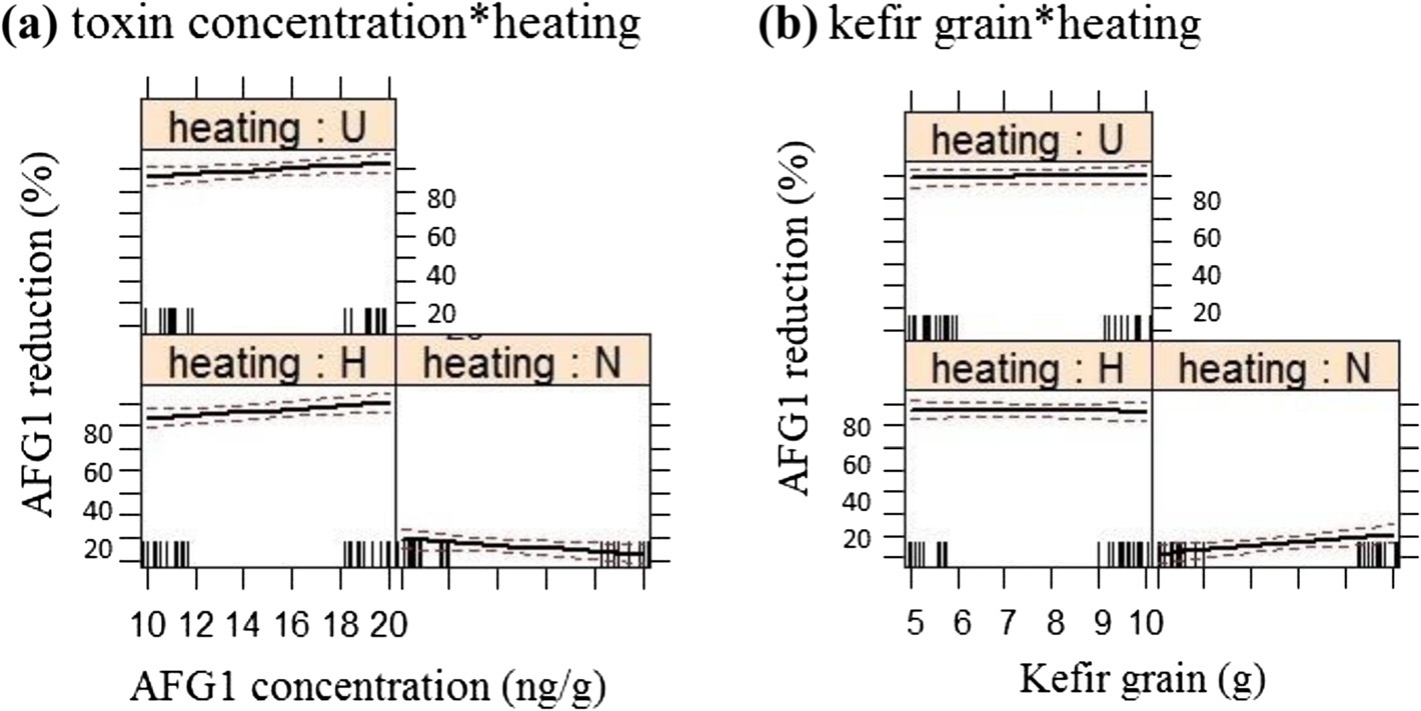
Effect plots of **(a)** toxin concentration; **(b)** kefir-grain level, on AFG1 reduction in three levels of heating treatments.

Figure 1 shows the positive effect of heat-treated kefir-grain (H and U) on AFG1 reduction. The results revealed that with an increase in toxin and/or kefir-grain concentration at least 80% of the AFG1 removed under heat-treated conditions. Several studies also reported that heat-treatment of the microorganism increased aflatoxin reduction, due to physical adsorption of the aflatoxin molecule to the cell wall components of the microorganisms [23,30-33]. However, these results were different in comparison with other reported studies which did not find any significant differences in the reduction of toxin by heat-treated microorganisms [21,34-36].

In optimization step, because of the economic aspect which was related to the need for less energy consumption in the H condition, kefir-grain treated with 70°C were used.

### Optimization and modelling of AFG1 reduction

A CCD for the variables [AFG1 concentration (X_1_), kefir-grain level (X_2_), contact time (X_3_) and incubation temperature (X_4_)], each at five levels with seven replicates at the centre point (to account for pure internal error and check the adequacy of curvature which offered by response), was applied for the optimization of AFG1 reduction using H-treated-kefir grain. The design experiments for optimization using RSM are shown in Table 1.

In this stage, the concentration of spiked-AFG1 (X_1_) applied in levels of 25 ng/g and below it (Table 3). This range was used because of the established MRL set for AFT in pistachio nuts (Iranian standard regulation = 15 ng/g and European Union legislation = 10 ng/g) [6,7]. The ranges of X_2_, X_3_ and X_4_ were extended based on the experimental design utilized in our current investigation (Table 3).

**Table 3:** **Central composite design levels of the most effective factors on AFG1 reduction**

**Factor**	**Level**				
	**-α**	**Low**	**Center**	**High**	**+α**
X_1_	5	10	15	20	25
*X*_2_	2.5	5.0	7.5	10.5	12.5
X_3_	0	2	4	6	8
X_4_	20	30	40	50	60

Note: (X_1_) toxin concentration, (X_2_) kefir grain level, (X_3_) contact time, and (X_4_) incubation temperature.

-α and + α are cube point levels to obtain a wider prediction space of factors.

Statistical parameters obtained from ANOVA were significant (Table 4). The ANOVA results indicated the adequacy of significant variables in the model. The lack-of-fit test was insignificant (*p* = 0.422). The mathematical model for response (AFG1 reduction) is shown in equation 1:

1 $$ Y=91.109+1.948{X}_1+0.572{X}_2+1.258{X}_3+0.05{X}_4+1.35{X}_2{X}_4\hbox{--}\;1.712{X}_3{X}_4 $$

where, *Y* is the measured response of AFG1 reduction and *Xi* is the coded independent variables (*X*_*1*_ = AFG1 concentration, *X*_*2*_ = kefir-grains level, *X*_*3*_ = contact time and *X*_*4*_ = incubation temperature).

**Table 4:** **Analysis of variance and coefficient estimates for model**

**Source of variation**	**Degree of freedom**	**Sum of squares**	**Mean square**	***F*** **-value**	***P*** **-value**
Linear	4	137.032	34.258	6.041	0.001
Interaction (X_2_:X_4_)	1	29.16	29.16	5.142	0.032
Interaction (X_3_:X_4_)	1	46.923	46.923	8.275	0.008
Residuals	24	136.088	5.67		
Lack of fit	18	107.232	5.957	1.238	0.422
Pure error	6	28.856	4.809		
**Term**	**Estimate**	**Standard Error**	***t*** **-value**	***P*** **-value**	**Significant**
Intercept	91.109	0.427	213.028	0.000	0.001
X_1_	1.948	0.486	4.008	0.000	0.001
X_2_	0.572	0.486	1.177	0.250	
X_3_	1.258	0.486	2.588	0.016	0.05
X_4_	0.05	0.486	0.102	0.918	
X_2_:X_4_	1.35	0.595	2.267	0.032	0.05
X_3_:X_4_	−1.712	0.595	−2.876	0.008	0.01

Note: (X_1_) toxin concentration; (X_2_) kefir grain level; (X_3_) contact time; (X_4_) incubation temperature.

P-value: 0.0004.

Table 4 and Equation 1 show the most significant factors and interactions that affected the response. As shown in Table 4, there was a positive relationship between AFG1 concentration and its reduction (*p* < 0.001). This result was confirmed by several studies which reported a linear relationship between toxin concentration and its removal by microorganisms [21,23].

**Figure 2:**
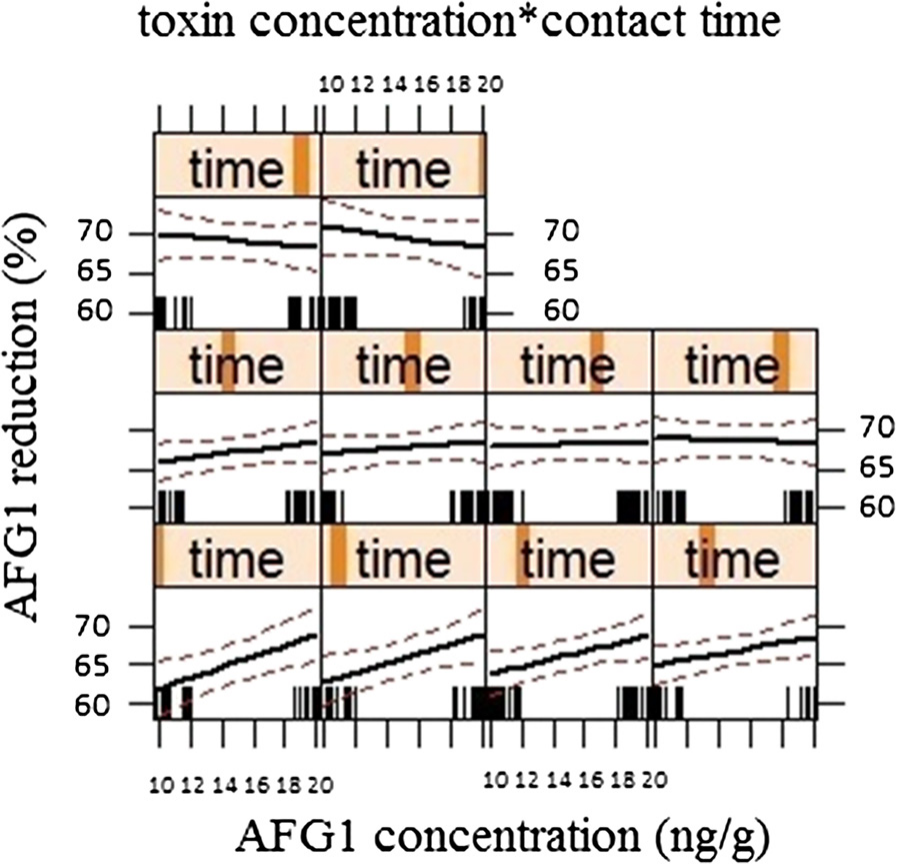
Effect plot of the model equation fitted to the interaction between AFG1 concentration and contact time (from 0 to 8 h).

The Effect plot of the model equation fitted to the interaction between toxin concentration and contact time shows in Figure 2. Contact time has a positive significant effect on response (*p* < 0.05) and AFG1 removal increased with increase in contact time. However, this result differs from those of other reports who showed that the efficiency of detoxification is not time-dependent [21,32,33,37]. This may be due to the symbiotic mixture of microbial association in kefir-grain. Hence, further studies need to demonstrate this opinion.

**Figure 3:**
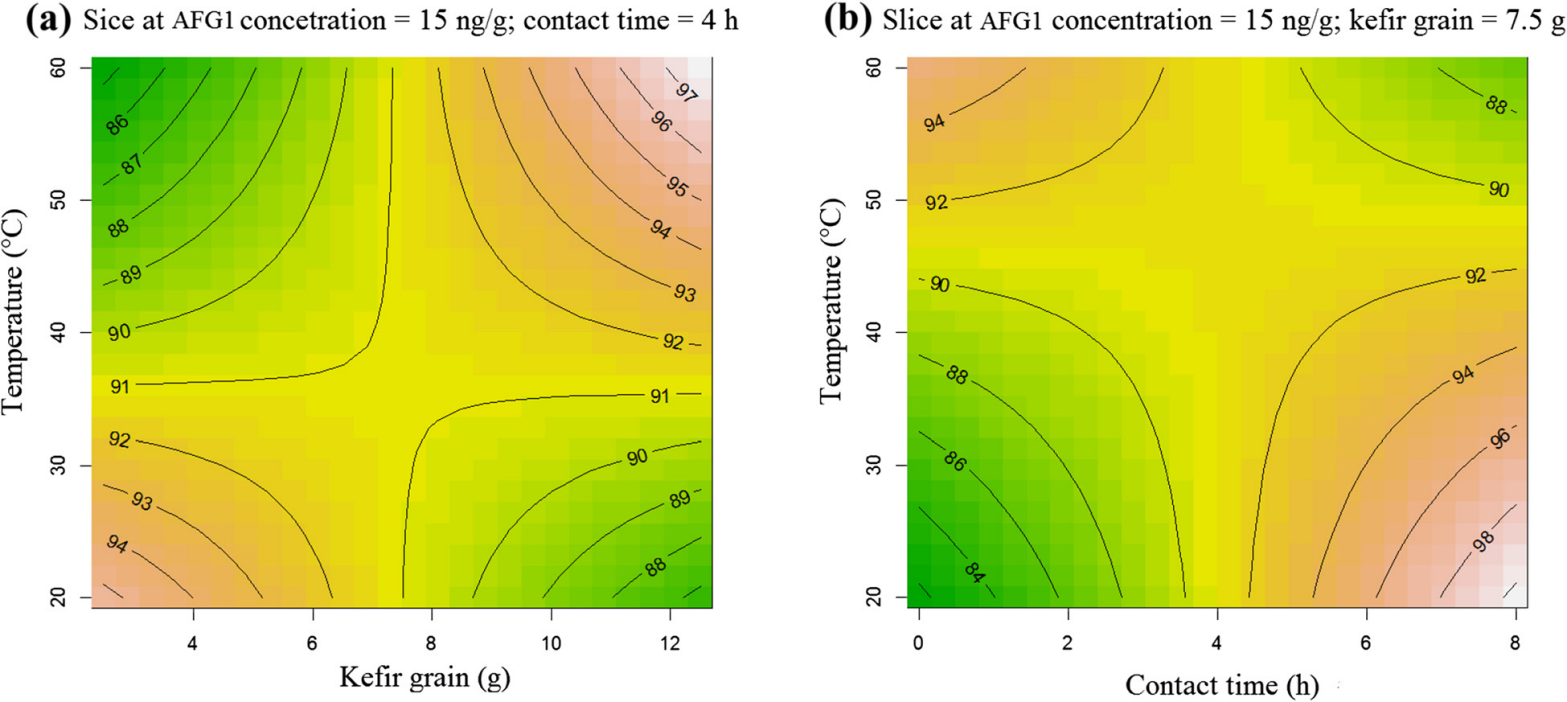
Contour plots of the model equation fitted to the interaction between: **(a)** kefir grain level and incubation temperature; **(b)** contact time and incubation temperature, on AFG1 reduction.

In this study, a significant interaction between X_2_-X_4_ (*p* < 0.05) and X_3_-X_4_ (*p* < 0.01) were observed (Table 4). Figure 3 shows contour plots of the relation between factors and response by holding all other factors at fixed levels. When kefir-grain concentrations was low (2–5 gr), AFG1 elimination decreased with increasing incubation temperature up to around 60°C. Also, the plot shows that using 7–8 gr kefir-grain, the percent of AFG1 reduction was constant (91%) and increasing the temperature did not effect on response. The best reduction was in the lowest concentration of kefir-grain together with minimum temperature or in the highest concentration of kefir-grain associated with the maximum temperature (Figure 3a)

.

Figure 3(b) shows that AFG1 elimination increased with increasing contact time up to 6–8 h at minimum temperature (20–30°C). However, the reduction in high temperatures (50–60°C) was fast and required only a short period of time (94% reduction in 0–2 h). It could be concluded that incubation temperature might be responsible for increasing the toxin–microorganism binding capacity.

The optimum conditions for the highest AFG1 reduction (96.8%) were predicted by the model as follows: toxin concentration = 20 ng/g, kefir-grain level = 5 gr (equal to 10% pistachio past sample), contact time = 6 h, and incubation temperature = 30°C.

### Verification of the optimal condition

In order to examine the predictability of the model and statistically validate the optimal conditions, agreement between the experimental (observed) and predicted value was investigated. Thus, the experimental values of the AFG1 reduction were obtained in six optimal condition replicates (mean = 94.54 ng/g) and predicted responses calculated by equation 1 (96.7%) compared using one sample *t*-test [38]. The *p-*value for one sample *t*-test was 0.15, indicating that there was no significant difference between predicted amounts fitted by the model and the experimentally obtained value (*p* > 0.05).

The final step in the present study was to check the validation of the method by regression analysis under optimal conditions in six replications. The correlation coefficient (*R*^*2*^) was 97.04, which indicated that only 2.69% of the experimental value was not explained by the optimal conditions of the model. This indicates that there is a high fit degree between the value observed in the experiment and the value predicted by the model.

## Conclusion

This study has provided the first evidence to demonstrate that kefir-grains have a significant effect on AFG1 decontamination from pistachio nuts. The second approach in this work was to investigate the effect of four independent variables including toxin concentration, kefir-grain level, contact time and incubation temperature on AFG1 reduction using a sequential optimization strategy (full factorial design followed by central composite design) and predict a model for optimizing the response. The results showed that both pre-heated kefir-grains at H (70°C) and U (110°C) conditions caused a significant decrease in AFG1 in contaminated pistachio samples. The amount of AFG1 elimination was dependent on toxin concentration (*p* < 0.001) and contact time (*p* < 0.05).

The optimization part of the study indicated that maximum AFG1 reduction (96.8%) would be obtained in toxin concentration at 20 ng/g, KG level at 10%, contact time at 6 h and an incubation temperature at 30°C. This result mentioned that even in low level of kefir-grain (10%) the amount of toxin removed was high (up to 96%), suggesting that a high efficiency of microorganisms existing in kefir-grain. Consequently, the model was validated statistically and practically under optimal conditions. Six replicated experiments were conducted under optimal conditions. There was a high fit degree between experimental results and values predicted by the model (*R*^*2*^ = 97.04).

In conclusion, the results of our investigation showed that an optimized biological detoxification method using kefir-grains treated in 70°C have good accuracy and would be suitable for routine AFG1 elimination in pistachio. Consequently, the offered model can be used to predict AFG1 reduction and could be utilize to develop a strategy for reducing toxin bioavailability in pistachio nuts.
